# International Medical Graduates in the US Plastic Surgery Residency: Characteristics of Successful Applicants

**Published:** 2018-11-27

**Authors:** George Kokosis, Angelo A. Leto Barone, Michael J. Grzelak, Sara Alfadil, Edward H. Davidson, Scott Lifchez, Amir H. Dorafshar

**Affiliations:** ^a^Department of Plastic and Reconstructive Surgery, The Johns Hopkins Hospital, Johns Hopkins School of Medicine, Baltimore, Md; ^b^Division of Plastic and Reconstructive Surgery, Albert Einstein College of Medicine, Montefiore Medical Center, Bronx, NY

**Keywords:** international medical graduate, plastic surgery match, integrated plastic surgery match, independent plastic surgery match, plastic surgery residency

## Abstract

**Objectives:** Within the United States, plastic surgery is a difficult field to match into for both US and international medical graduates. While the number of available residency positions has grown in recent years, this has not been mirrored by an equal increase in the number of international medical graduates who match. Furthermore, there are few reliable resources to guide international medical graduates who are interested in matching into US-based programs, so the process is often even more difficult and unpredictable than for US applicants. **Methods:** An anonymous survey was distributed electronically to international medical graduates who successfully matched into independent and integrated US plastic surgery residency programs. The survey assessed qualities such as medical school performance, test scores, research experience, and other relevant applicant information, and χ^2^ analysis was done to compare the survey results for integrated and independent track international medical graduates. **Results:** International medical graduates who successfully match tend to rank high and score well in their medical school classes, score between 230 and 250 on USMLE step 1 and 2CK tests, and have a mean of 2 years of research experience before applying to the match. International medical graduates in the independent track tend to have higher step 1 scores, whereas international medical graduates in the integrated track tend to have more research experience and additional nonmedical degrees. **Conclusions:** This is a survey-based overview that describes the characteristics of successfully matched international medical graduates. Limitations of this study include the inability to identify and survey the unsuccessful applicants as well as poor response rate of the successful candidates in the independent pathway who successfully matched.

The field of plastic surgery has a competitive residency matching process, with 26% of US Seniors and 80% of international medical graduates (IMGs) and non-US Senior applicants (all applicants other than fourth-year students in US allopathic schools) failing to match into integrated programs in 2017.^1^ Furthermore, according to step 1 scores, matching in plastic surgery has become much more competitive in recent years and the average step 1 score of incoming plastics residents has risen from 233 in 2006 to 247 in 2017.^2^ While the field is currently undergoing a rapid expansion and admitting more residents each year,^1^^,^^3^ the number of successfully matching IMGs has been relatively stagnant in recent years. In fact, according to National Resident Matching Program (NRMP) data, between 2008 and 2012, the mean number of residents accepted into integrated plastic surgery residencies was 92 and the mean number of IMGs admitted was 4 per year. Between 2013 and 2017, while the mean number of accepted residents grew to 144, the mean number of IMGs admitted remained at 4 per year.^1^ The number of IMGs accepted through the independent track is only slightly higher, and according to the SF Match data for 2016, 7 IMGs matched out of a total of 70 positions offered.^3^ Upon reviewing the match data and observing these paltry numbers, it is not surprising that IMG applicants consider the task of matching a daunting endeavor. Furthermore, while data have been published on what makes a US graduate likely to succeed in both the integrated and independent matches, including Alpha Omega Alpha membership, step 1 score, and publications,^4^^-^9 very little information is available to IMG applicants regarding the application elements that make a successful IMG profile.

In this study, surveys were sent to IMG applicants who successfully matched into US plastic surgery programs, whether independent or integrated, in an effort to elucidate the characteristics that build a successful IMG application and provide a resource to these applicants. Unfortunately, the surveying of unsuccessful IMG applicants was not feasible because the records of these applicants are not available through an official Web site.

## METHODS

An anonymous survey was sent to all US plastic surgery training programs to be distributed to the IMGs who are currently enrolled or recently graduated from the programs between 2009 and 2016. The survey was evaluated and approved by the Johns Hopkins Medicine institutional review board (IRB00115476). Recipients received a written explanation of the goals of the study, and their consent to participate in the study was assumed upon completion and submission of the voluntary survey. The respondents’ demographics, including the type of residency path (integrated or independent), type of medical school attended, years of medical school required to graduate, location of the medical school, and information about prior surgical training, were obtained. Questions to evaluate academic achievements, clinical background, number of publications and grants, years of research completed before applying, and board scores were also included. Both NRMP and SF Match official data were used to validate the pool of applicants for the integrated and independent tracks in order to calculate response rates. The data were collected, consolidated, and analyzed separately for the independent and integrated IMG groups and then χ^2^ analysis was performed to compare the 2 groups. Statistical significance was assessed at *P* < .05.

## RESULTS

### Response rate

Of 87 surveys sent out, 30 were completed. Twenty-six surveys were sent out to residents in the integrated track and 19 responses were received, for a response rate of 73%, and 61 surveys were sent to residents in the independent track and 11 responses were received, for a response rate of 18% (Table 1).

### Performance in medical school

**Figure 1:**
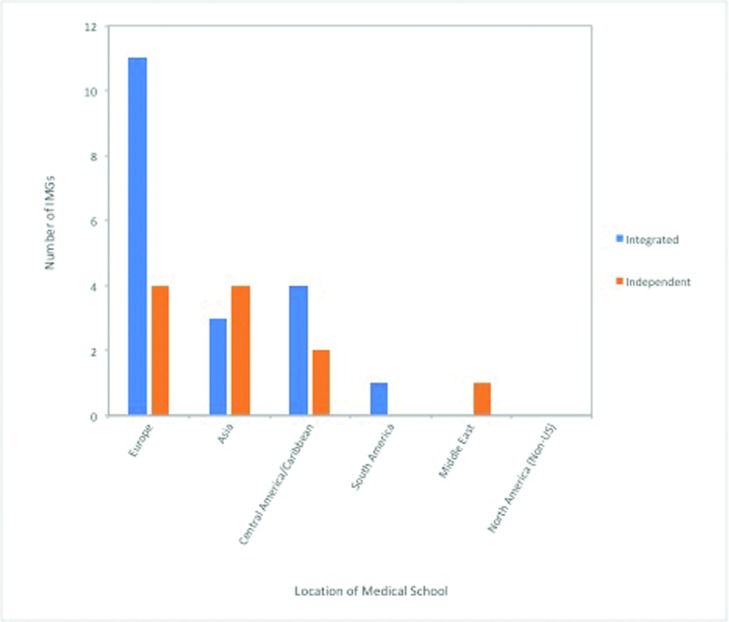
Characterizing IMG medical school location. Integrated and independent IMGs, *P* = .374. IMG indicates international medical graduate.

Successfully matched IMGs performed very well in medical school, with 37% of integrated IMGs and 18% of independent IMGs ranking in the top 1% of the class, with 37% of integrated IMGs and 45% of independent IMGs receiving honors in more than 90% of examinations and rotations. Of note, 37% of integrated IMGs and 18% of independent IMGs had a second degree (PhD, master's, other) in addition to their medical degree. Figure 1 delineates the location of IMG medical schools.

### Research background

**Figure 2:**
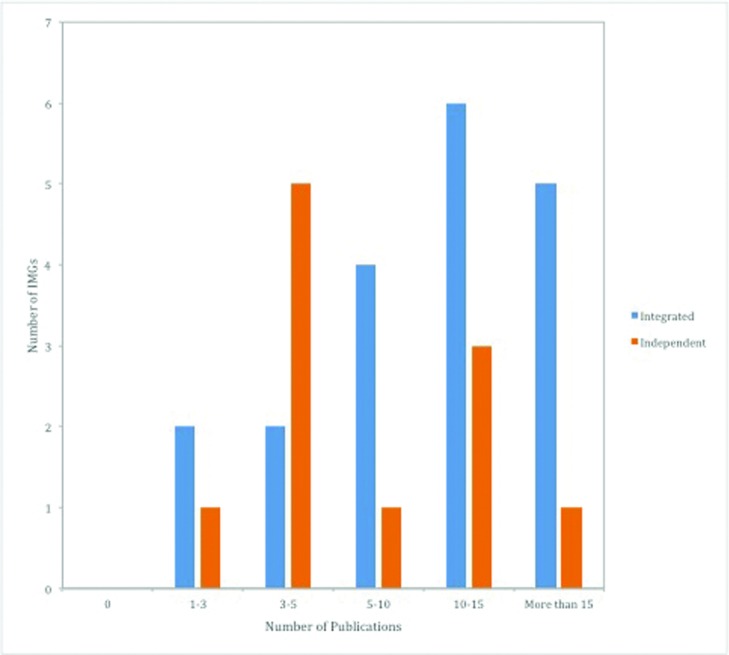
Characterizing research output among IMGs before applying to the NRMP or SF Match, *P* = .255. IMG indicates international medical graduates; NRMP, National Resident Matching Program.

Integrated IMGs had research-heavy backgrounds, with half of them having at least 3 years of research experience before matching and almost two-thirds having 10 or more publications (Fig 2). Furthermore, half of them reported receiving grants for their research as a Principal Investigator (PI) or Co-PI. In contrast, all independent IMGs had either 1 or 2 years of research experience, they most commonly had 3 to 5 publications, and none received grants as a PI or Co-PI.

### Test scores

**Figure 3:**
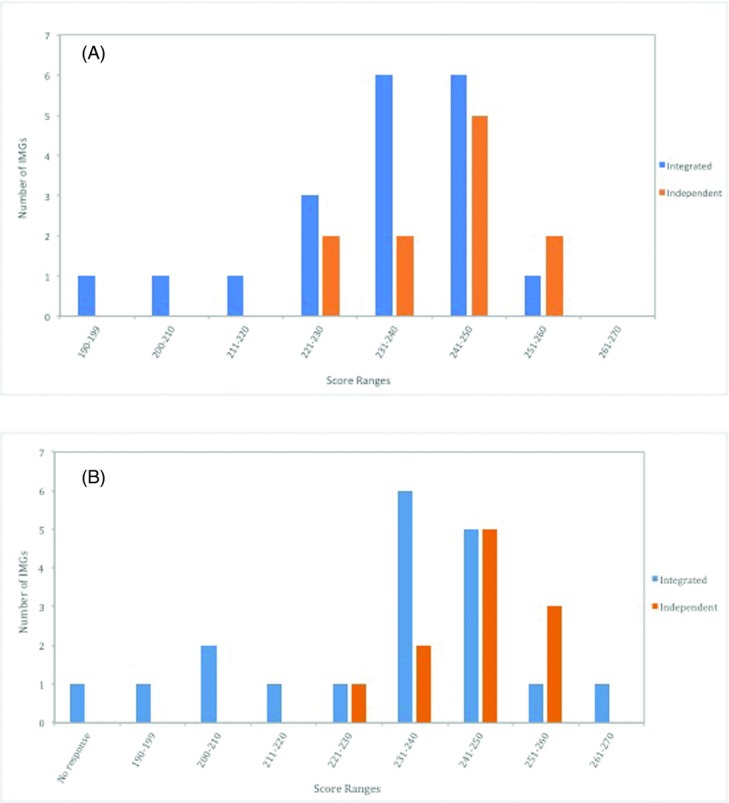
Characterization of IMG USMLE step 1 and 2CK test results. (a) USMLE step 1 score ranges for integrated and independent IMGs who matched into plastic surgery, *P* = .709. (b) USMLE step 2CK score ranges for integrated and independent IMGs who matched into plastic surgery, *P* = 0. IMG indicates international medical graduates.

Almost two thirds of integrated IMGs scored between 231 and 250 on USMLE step 1 and step 2 (Fig 3). Interestingly, a third of integrated IMGs reported lower-tier step 1 scores between 190 and 230. The most common step 1 and step 2 scores for independent IMGs, meanwhile, were between 241 and 250.

### Results of the match

**Figure 4:**
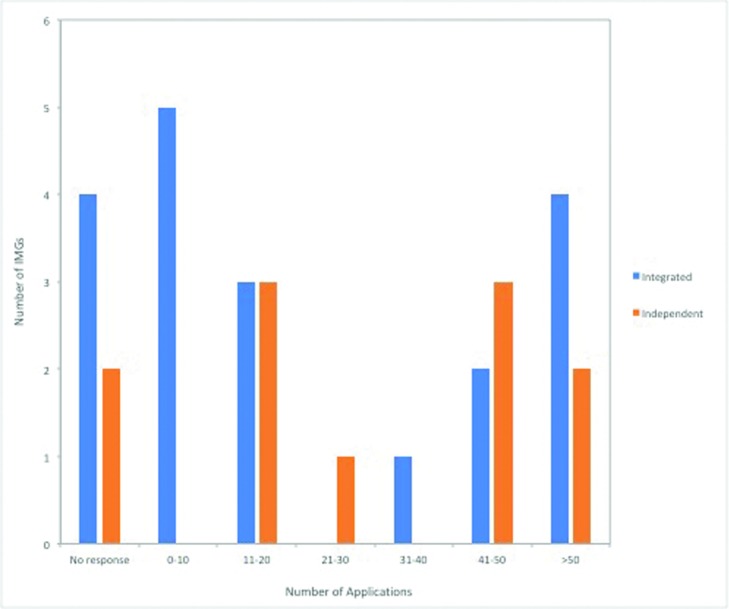
Number of programs each IMG applied to during the match, *P* = .237. IMG indicates international medical graduates.

Integrated and independent IMGs applied to programs in a bimodal distribution, with one third applying to a low number of programs (1-20) and one third applying to a high number of programs (>40) (Fig 4). As for the results of the match, two thirds of the integrated respondents were successful in matching into plastic surgery in their first application cycle. For both integrated and independent IMGs, more than half of them matched into their highest-ranked program (Table 1).

## DISCUSSION

Historically, it has been very difficult for IMGs to match into US plastic surgery programs, and while there are a variety of factors involved, emerging data such as this study are making it clear that this historical trend is not based on differences in academic achievement. As many in the medical community suggest, IMGs who graduate from rigorous international schools perform as well as their US counterparts on the USMLE examinations and deliver as good, if not better, care to their patients.^10^

Some of the obstacles that may be contributing to the trend of low IMG acceptance include concerns regarding overall competency and breadth of training of IMGs, concerns that IMG acceptance will lower the program's prestige or ACGME status, and issues with visa sponsoring and its related requirements.^11^^-^13 Historically, concerns regarding the competency and training of IMGs were common among US residency programs. The literature regarding this topic continues to suggest, however, that IMGs perform at least as well as their US graduate counterparts in clinical and academic settings. For surgical outcomes in particular, a recent study showed that there is no difference between IMG and US graduate surgeons in their rates of surgical complications, mortality, or length of stay.^14^ Related to the concern of clinical competency is the concern regarding ACGME competency and accreditation. A study comparing the ACGME competency of IMGs and US graduates found that there are no differences in overall ACGME competency and medical knowledge between the groups, although IMGs tended to score lower in questions regarding US health care economics.^13^ Furthermore, the ACGME-I program has been created to ensure that IMGs trained at accredited international institutions have similar ACGME competency to US graduates.^15^ It is therefore fair to state that once these individuals (IMGs) enter a US residency program, no bias appeared to be exhibited toward them, whether during job searches or by important professional societies such as the ASPS or even the American Board of Plastic Surgeons.

Finally, another concern regarding the acceptance of non-US citizen IMG applicants is the difficulties IMGs may have in attaining visa sponsorship. J1 visas represent the most common visa category offered to newly matched IMGs due to the ease and rapidity of processing, as well as its low cost compared with other visa categories such as H1B and O1 visas. J1 visas, however, have the major drawback of requiring the IMG physicians to return to their home countries for 2 years at the end of training or to serve in an underserved area for 3 years. Furthermore, in recent years, additional restrictions have been placed on visas and have impeded the ability of many IMGs to obtain visas. Legal action regarding the restrictions on J1 and H1B visas may lower these obstacles in the upcoming years, but in the meantime, obtaining visa status for IMGs may present a challenge to US institutions.^12^ In summary, while concerns over diminished clinical, surgical, and ACGME competencies appear to be unfounded reasons to deny acceptance of IMGs into US plastics residencies, the visa status of IMG applicants may represent a significant hurdle to some US programs.

In this study, we found additional evidence to support the competency and academic excellence of IMG applicants. We found that both integrated and independent IMGs perform well on their USMLE examinations, excel on their clinical rotations, and have strong research backgrounds. The emerging trends in this study indicate that independent IMGs in plastics have stronger USMLE examination scores while integrated IMGs have stronger research backgrounds and additional degrees. Although it is difficult to discern why these differences exist between the 2 tracks, one explanation is that integrated students may commit to plastics earlier on, focus on garnering years of research in the field, and ultimately score lower on the USMLE tests because they have an extended research-focused hiatus between the exposure to the material in medical school and the examinations. Independent students, on the contrary, may be undecided on a particular specialty and therefore opt to take the USMLE examinations immediately after graduating from medical school, forgo extensive research experiences, and enter directly into general surgery residencies.

The purpose of this study was to evaluate the characteristics of IMGs who successfully matched into plastic surgery residency programs. Although we did not perform a direct comparison with the matched US graduates, the accolades of the IMGs compare similarly to their counterparts when examining the literature. Tadisina et al^16^ discussed the increasing accomplishments of US graduates matching into a plastic surgery residency program from 2009 to 2014. The number of publications ranged from 8 to 12 per applicant, and mean USMLE step 1 scores were 245 and step 2 scores ranged from 245 to 250. These numbers have increased over the years when compared with older studies,^17^ reflecting the increasing competitiveness in the match. However, they are similar to our findings for IMGs who successfully complete the match process. These findings suggest that IMGs who successfully match have proven themselves to be competitive when compared with US graduates.

It is also important to discuss how the trends of IMGs in plastic surgery compare with the trends in other surgical fields. For example, while the percentage of IMGs in plastic surgery has been relatively stagnant, the percentage of IMGs accepted into general surgery has been increasing.^18^ Similarly to plastic surgery IMGs, orthopedic surgery IMGs have strong research backgrounds and have twice the number of research experiences as their US Senior counterparts at the time of the match.^19^ Finally, according to 2017 NRMP data for surgical specialties, plastic surgery is an exceptionally competitive surgical residency for IMGs, with only 20% of IMG and non-US Senior applicants successfully matching. This makes plastics more competitive for IMGs than orthopedic surgery, neurosurgery, and vascular surgery, and indeed only thoracic surgery was a more difficult surgical residency for IMGs to match into.^1^

Although the study succeeded in elucidating a profile of the successful IMG, an important point to address is the general lack of statistical significance in differences between independent and integrated residents. This lack of statistical significance is most attributable to the small number of participants (n = 30) in the study, and one unavoidable cause is that there are few IMG plastics residents in the United States. The low response rate from independent IMG residents (18%) also contributed to the low number of participants. Another limitation of the study is that we were unable to identify and send surveys to unsuccessful IMG applicants, as confidentiality precludes identifying those applicants and surveying them. Because of this limitation, we cannot assess the baseline characteristics of all IMG applicants and therefore we cannot determine which characteristics seem most correlated with differentiating a successful application from an unsuccessful one.

## CONCLUSION

The main goal of this study was to elucidate the profiles of IMGs who have successfully matched into US plastic surgery programs, and key findings include the extensive research experience of integrated IMGs and the strong USMLE examination performances of independent IMGs.

Along with highlighting the qualities that tend to make IMGs successful in the plastic surgery residency match, this study reveals that, despite the stigma that surrounds these candidates, many IMGs are strong, well-rounded, and competitive applicants who demonstrate a high level of drive and devotion to the advancement of the field of plastic and reconstructive surgery.

## Figures and Tables

**Table 1:** Independent and integrated IMG plastic surgery resident characteristics*

	Integrated IMGs	Independent IMGs	
Characteristics	(units: # of residents)	(units: # of residents)	*P*
Response rate	19/26 (73%)	11/61 (18%)	
Honors in classes and rotations			
>90% of class	7/19 (37%)	5/11 (45%)	.723
70%-90% of classes	6/19 (32%)	2/11 (18%)	
Class rank			
*Top 1%*	7/19 (37%)	2/11 (18%)	.206
*Top 5%*	4/19 (21%)	6/11 (55%)	
Publications			
*5-10*	4/19 (21%)	1/11 (9%)	.255
*10-15*	6/19 (32%)	3/11 (27%)	
*>15*	5/19 (26%)	1/11 (9%)	
Research grants	8/19 (42)%	0/11 (0%)	**.012**^b^
USMLE step 1			
*190-199*	1/19 (5%)	0/11 (0%)	.709
*200-210*	1/19 (5%)	0/11 (0%)	
*211-220*	1/19 (5%)	0/11 (0%)	
*221-230*	3/19 (16%)	2/11 (18%)	
*231-240*	6/19 (32%)	2/11 (18%)	
*241-250*	6/19 (32%)	5/11 (45%)	
*251-260*	1/19 (5%)	2/11 (18%)	
USMLE step 2CK			
*190-199*	1/19 (5%)	0/11 (0%)	.461
*200-210*	2/19 (11%)	0/11 (0%)	
*211-220*	1/19 (5%)	0/11 (0%)	
*221-230*	1/19 (5%)	1/11 (9%)	
*231-240*	6/19 (32%)	2/11 (18%)	
*241-250*	5/19 (26%)	5/11 (45%)	
*251-260*	1/19 (5%)	3/11 (27%)	
*261-270*	1/19 (5%)	0/11 (0%)	
Research years			.242
*1*	4/19 (21%)	3/11 (27%)	
*2*	5/19 (26%)	4/11 (36%)	
*3*	7/19 (37%)	0/11 (0%)	
*>3*	2/19 (11%)	0/11 (0%)	
Number of applications sent out			
0-10	5/19 (26%)	0/11 (0%)	.237
11-20	3/19 (16%)	3/11 (27%)	
21-30	0/19 (0%)	1/11 (9%)	
31-40	1/19 (5%)	0/11 (0%)	
41-50	2/19 (11%)	3/11 (27%)	
Matched at first choice	11/19 (58%)	5/11 (55%)	.09
Matched at second choice	3/19 (16%)	0/11 (0%)	.09

^*^IMG indicates international medical graduate.

^†^The value in bold indicates significant statistical difference (*P* < .05).
